# Asia-Pacific expert consensus on robotic-assisted hip and knee arthroplasty

**DOI:** 10.1186/s42836-026-00427-1

**Published:** 2026-08-13

**Authors:** Guoqiang Zhang, Yixin Zhou, Yunsu Chen, Nicolaas C. Budhiparama, Chun Hoi Yan, Seung Jae Lim, Lincoln Ming Han Liow, Nobuhiko Sugano, Andrew Tang, Hong Cai, Dirk van Bavel, Wei Chai, Ping Keung Chan, Xinyu Fang, Eryou Feng, Henry Fu, Takafumi Hiranaka, Zongke Zhou, Kang-il Kim, Andrew Kurmis, Huiwu Li, David Liu, Xianzhe Liu, Sébastien Lustig, Xinzhan Mao, Tomoyuki Matsumoto, Ming Ni, Michael Ong, Sang Eun Park, Wenwei Qian, Hongyi Shao, Chandeep Singh, Hao Tang, Yukihide Minoda, Haobo Wu, Jie Xie, Seng-Jin Yeo, Xiaogang Zhang, Aree Tanavalee, Li Cao, Myung Chul Yoo, Yan Wang

**Affiliations:** 1 Department of Orthopaedic Surgery, First Medical Center, Chinese PLA General Hospital, Beijing, 100853 China; 2 Department of Orthopaedic Surgery, Fourth Medical Center, Chinese PLA General Hospital, Beijing, 100048 China; 3 Department of Orthopaedic Surgery, Beijing Jishuitan Hospital, Capital Medical University, Beijing, 100035 China; 4 Department of Orthopaedics, Shanghai Sixth People’s Hospital, Shanghai Jiao Tong University School of Medicine, Shanghai, 200030 China; 5 Department of Orthopaedic and Traumatology, Faculty of Medicine, University of Airlangga, Surabaya, 60131 Indonesia; 6 Department of Orthopaedics, Leiden University Medical Centre, Leiden, 2333 ZA The Netherlands; 7 Nicolaas Institute of Constructive Orthopaedic Research & Education Foundation for Arthroplasty & Sports Medicine at Medistra Hospital, Jakarta, 12950 Indonesia; 8 Department of Orthopaedics and Traumatology, Gleneagles Hospital Hong Kong, Hong Kong SAR, China; 9 Department of Orthopaedic Surgery, Samsung Medical Center, Sungkyunkwan University School of Medicine, Seoul, South Korea; 10 Department of Orthopaedic Surgery, Singapore General Hospital, Singapore, 169865 Singapore; 11 Department of Orthopaedic Medical Engineering, Osaka University Graduate School of Medicine, 2-2 Yamadaoka, Suita, 565-0871 Japan; 12 Musculoskeletal Institute, Epworth Healthcare, Melbourne, VIC 3121 Australia; 13 Department of Orthopedics, Peking University Third Hospital, Beijing, 100191 China; 14 Department of Orthopaedic Surgery, St Vincent’s Hospital, Melbourne, Australia; 15 Department of Orthopaedics and Traumatology, School of Clinical Medicine, The University of Hong Kong, Hong Kong SAR, China; 16 Department of Orthopedic Surgery, the First Affiliated Hospital, Fujian Medical University, Fuzhou, 350005 China; 17 Department of Orthopaedics, Fujian Medical University Union Hospital, Fuzhou, 350001 China; 18 Department of Orthopaedic Surgery and Joint Surgery Centre, Takatsuki General Hospital, Takatsuki, Japan; 19 Department of Orthopedic Surgery, Orthopedic Research Institute, West China Hospital, Sichuan University, Chengdu, China; 20 Department of Orthopaedic Surgery, Kyung Hee University Hospital at Gangdong, Seoul, 05278 Korea; 21 Department of Orthopaedic Surgery, School of Medicine, Kyung Hee University, Seoul, 02447 Korea; 22 Discipline of Medical Specialties, University of Adelaide, Adelaide, SA Australia; 23 Department of Orthopaedic Surgery, Lyell McEwin Hospital, Elizabeth Vale, SA Australia; 24 College of Medicine & Public Health, Flinders University, Bedford Park, SA Australia; 25 Orthopaedic, Shanghai Jiao Tong University School of Medicine Affiliated Ninth People’s Hospital, Shanghai, China; 26 The Gold Coast Centre for Bone and Joint Surgery, 14 Sixth Avenue, Palm Beach, Gold Coast, 4224 Australia; 27 Department of Orthopedic Surgery, Wuhan Union Hospital, Tongji Medical College, Huazhong University of Science and Technology, Wuhan, 430022 China; 28 Orthopaedics Surgery and Sports Medicine Department, FIFA Medical Center of Excellence, Croix-Rousse Hospital, Lyon University Hospital, 69004 Lyon, France; 29 Department of Orthopedics, The Second Xiangya Hospital of Central South University, Changsha, 410011 China; 30 Department of Orthopaedic Surgery, Graduate School of Medicine, Kyoto University, 54 Shogoin Kawaharacho, Sakyo, Kyoto, 606-8507 Japan; 31 Department of Orthopaedics & Traumatology, Faculty of Medicine, The Chinese University of Hong Kong, Hong Kong SAR, China; 32 Department of Orthopedic Surgery, Gangnam St. Peter’s Hospital, Seoul, 06275 Republic of Korea; 33 Department of Orthopedic Surgery, Peking Union Medical College Hospital, Beijing, 100730 China; 34 Max Institute of Musculoskeletal Sciences & Orthopaedics, New Delhi Gurugram, India; 35 Department of Orthopedic Surgery, Osaka Metropolitan University, Osaka, Japan; 36 Department of Orthopaedics, the Second Affiliated Hospital of Zhejiang University School of Medicine, Hangzhou, 310009 China; 37 Department of Orthopedics, The First Affiliated Hospital, Zhejiang University School of Medicine, Hangzhou, 310003 Zhejiang China; 38 Department of Orthopaedics, First Affiliated Hospital of Xinjiang Medical University, Urumqi, 830054 China; 39 Department of Orthopaedics, Faculty of Medicine and King Chulalongkorn Memorial Hospital, Chulalongkorn University, Bangkok, 10330 Thailand

**Keywords:** Robotic-assisted surgery, Total knee arthroplasty, Total hip arthroplasty, Consensus, Arthroplasty Society in ASIA, Delphi method

## Abstract

**Background:**

Robotic-assisted total joint arthroplasty (rTJA) has witnessed rapid adoption across the Asia-Pacific region. However, practice heterogeneity and conflicting evidence regarding its clinical value necessitate standardized guidance. Therefore, this expert consensus aims to establish consensus-based recommendations to guide and standardize the clinical application of rTJA.

**Methods:**

The Arthroplasty Society in Asia (ASIA) convened a panel of 45 experts. A modified Delphi technique was employed across three rounds. A systematic literature review informed the generation of consensus statements, which were finalized through anonymous voting. Consensus was defined as ≥ 75% agreement.

**Results:**

The panel reached consensus on 18 critical clinical questions spanning general principles, total hip arthroplasty, and total knee arthroplasty. Key consensus points include: (1) rTJA demonstrates superior accuracy in implant positioning and alignment restoration compared to manual techniques (98%); (2) current evidence does not yet support a definitive superiority in long-term survivorship or PROMs (77%); (3) robotic platforms are enabling technologies that make personalized alignment philosophies technically reproducible (100%); and (4) soft-tissue balancing should take precedence over rigid mechanical alignment targets in rTKA when conflicts arise(91%).

**Conclusion:**

This consensus statement provides a comprehensive framework for the application of robotic technology in hip and knee arthroplasty, emphasizing safety, precision, and the need for rigorous training.

## Background

Over the past decade, robotic-assisted total joint arthroplasty (rTJA) has seen a rapidly increasing integration into daily orthopaedic practice worldwide. The 2024 American Joint Replacement Registry (AJRR) Annual Report highlights this upward trajectory, noting that robotic assistance was utilized in 15.9% of primary TKA and 6.6% of elective primary THA procedures in 2023 [1]. Despite this widespread adoption, the technology’s integration into clinical practice has outpaced the establishment of standardized guidelines, resulting in significant practice heterogeneity [2]. Given the substantial accumulation of evidence and clinical experience over the past decade, a systematic synthesis of core controversies is now imperative to guide the responsible evolution of rTJA.

Currently, the field of robotic arthroplasty confronts three distinct categories of challenges:

First, the translation of surgical precision into tangible long-term clinical value remains the primary subject of scrutiny. While the superiority of robotics in reducing outliers and enhancing component placement accuracy is well-documented [3, 4], the critical question persists: does this submillimeter precision reliably translate into superior survivorship and safety? Evidence remains conflicting regarding whether rTJA significantly reduces the incidence of major complications compared to conventional manual techniques [2, 5–11]. Specifically, debate continues as to whether robotic assistance effectively lowers revision rates, minimizes infection risks, or reduces the incidence of dislocation in Total Hip Arthroplasty (THA). Furthermore, whether these technical advantages yield perceptible improvements in patient-reported outcome measures (PROMs) and satisfaction scores remains to be definitively established.

Second, fundamental debates persist regarding optimal technical paradigms and intraoperative philosophies. The enhanced capabilities of robotic systems have challenged traditional surgical dogmas, yet the “ideal” target remains elusive.

In Total Knee Arthroplasty (TKA), the availability of robotic precision has fueled a divergence in alignment philosophy. While robotic platforms facilitate the execution of individualized strategies—such as kinematic alignment (KA), restricted kinematic alignment (rKA), and functional alignment (FA)—marking a shift away from the strict adherence to neutral mechanical alignment (MA), consensus on the superior strategy is lacking [12–15]. Concurrently, validated thresholds for optimal soft-tissue tension and the resolution of conflicts between gap balance and bone resection remain undefined [16–18]

In Total Hip Arthroplasty (THA), robotic platforms provide the unique capability to dynamically assess spinopelvic mobility, offering a theoretical advantage in mitigating impingement risks through real-time simulation. Consequently, the traditional static “Lewinnek Safe Zone” is facing increasing challenges, with emerging research attempting to redefine “functional safe zones” tailored to individual patient dynamics rather than population averages [19].

Third, technological heterogeneity and economic feasibility require objective evaluation. The diversity of robotic platforms—ranging from image-based to image-less systems—introduces variability in learning curves, safety profiles, and specific complications such as pin-site fractures [20], complicating the generalization of clinical outcomes. Furthermore, the economic impact of rTJA remains debated. Given the significant capital and consumable costs, determining the cost-utility ratio and identifying specific implementation hurdles—particularly within the diverse healthcare landscapes of the Asia–Pacific region—are critical for defining the technology’s responsible application [21].

In summary, robotic-assisted arthroplasty is undergoing a profound paradigm shift—transitioning from the technical question of “how to achieve precision” to the philosophical question of “what is the correct target” and “is it worth the cost.” To navigate these uncertainties and establish a framework for current clinical practice, the Arthroplasty Society in Asia (ASIA) convened this international consensus meeting.

## Methods

### Study design and panel selection

This consensus statement was developed by the Arthroplasty Society in Asia (ASIA) utilizing a modified Delphi technique. To ensure methodological rigor, the organizational structure was explicitly defined:

Steering Committee: A core group comprising the editorial board and three designated lead experts. This committee oversaw the methodology, stratified the topics into three main domains (General, THA, and TKA), and ensured scientific rigor.

Stakeholders Group: The consensus panel consisted of 45 experts from 11 countries and regions, representing key regional practitioners. Selection criteria for these stakeholders were rigorously established based on academic publication records, leadership within professional societies, and high-volume caseloads in robotic-assisted total joint arthroplasty (rTJA).

Subgroup Panels: Subgroups were employed exclusively for delegated evidence synthesis and drafting. However, to maintain holistic consensus integrity, no isolated subgroup voting occurred; all 44 stakeholders participated in the final voting for all questions.

#### Round 1: item generation and evidence synthesis

The initial phase focused on item generation. The Stakeholders Group submitted critical issues requiring clarification via an open-ended survey. The proposed topics were stratified by the Steering Committee into the aforementioned three domains. Following a relevance ranking, specific questions were assigned to subgroup experts to conduct independent systematic reviews and draft initial recommendations. The Steering Committee performed a standardized review of all drafts.

#### Round 2: interim deliberation and refinement

A planned deviation from the classical Delphi method was introduced in this phase. While a standard Delphi relies purely on isolated, asynchronous, and anonymous iterative surveys, we conducted a synchronous hybrid meeting (combining online and offline participation). This modification was deemed necessary because pure written feedback is often insufficient to resolve complex, highly technical surgical controversies. During this round, the panel engaged in robust interactive discussion. Based on expert feedback and real-time debate, the wording of the statements was modified to resolve ambiguities.

#### Round 3: final voting and consensus definition

Following the deliberation phase, the final consensus meeting was convened. The entire Stakeholders Group cast anonymous votes on the finalized statements via an electronic system. Each recommendation offered three voting options: Agree, Disagree, or Abstain. Consensus was defined a priori based on strict criteria: a recommendation was considered to have achieved consensus only if ≥ 75% of the panelists voted “Agree” [22]. Recommendations failing to meet this threshold were recorded as lacking consensus and were excluded from the final guidelines.

### Statements of group 1: general principles in robotic-assisted arthroplasty

#### What are the fundamental components required to constitute a robotic-assisted surgical system for joint arthroplasty?

##### Recommendation

The robotic-assisted surgical system for joint arthroplasty should integrate these nine components: Robotic Arm, Central Control Computer, Optical Tracking System, Preoperative Planning Module, Intraoperative Registration System, Assistive Control Concepts, Specialized Tools and Cutting Blocks, Automatic Cutting Speed Control and Stop Functions, and Automated Matching Systems.

##### Delegate Vote

Delegate vote: Agree 95%, Disagree 2%, Abstain 3%.

##### Justification

This consensus outlines the essential elements required to ensure precise, consistent, and safe surgical performance. A comprehensive robotic-assisted surgical system typically comprises the following nine key components [23, 24]:

*Robotic Arm (Manipulator)*: Serving as the execution unit, the manipulator is responsible for precise intraoperative maneuvers. It generally consists of a dedicated medical robot or an adapted industrial robot possessing at least six degrees of freedom.

*Central Control Computer*: This processing unit analyzes imaging data acquired preoperatively or intraoperatively, facilitating surgical planning and providing real-time guidance.

*Optical Tracking System*: This component captures positional data from patient-mounted trackers, enabling the robot to execute accurate, real-time spatial orientation.

*Preoperative Planning Module*: Allowing for varying planning strategies, this module enables surgeons to formulate surgical plans tailored to patient-specific anatomy.

*Intraoperative Registration System*: Registration aligns the robotic coordinate system with the patient’s anatomical structures, a critical step for ensuring navigational accuracy throughout the procedure.

*Assistive Control Modalities*: Systems employ various control concepts. For example, haptic feedback or shared-control models allow manual guidance by the surgeon while the system actively restricts bone resection to predefined boundaries (boundary control).

*Specialized Instrumentation and Cutting Guides*: Certain systems utilize proprietary surgical instruments and cutting blocks designed to assist in precise osteotomy.

*Automatic Speed Control and Safety Stops*: Features such as real-time cutting speed adjustment and automatic stop mechanisms are integrated to enhance procedural safety and precision.

*Automated Matching Systems*: Specific platforms offer automated capabilities for implant sizing and trajectory adjustments during surgery.

Additionally, image-based systems typically necessitate detailed preoperative imaging, such as CT scans or long-leg radiographs, for planning. Conversely, imageless systems facilitate intraoperative planning without the requirement for preoperative radiological data.

#### What are the primary limitations hindering the widespread adoption of robotic technology in orthopedic surgery?

##### Recommendation

The universal adoption of robotic-assisted surgery is currently constrained by three primary factors: (1) the paucity of high-quality evidence demonstrating superior cost-effectiveness; (2) substantial systemic heterogeneity across nations regarding legal frameworks, regulatory pathways, and healthcare economics; and (3) barriers in standardized skills acquisition and continuing medical education. A concerted, multi-stakeholder strategy is imperative to address these impediments.

##### Delegate Vote

Delegate vote: Agree 93%, Disagree 2%, Abstain 5%.

##### Justification

The widespread adoption of robotic-assisted surgery in orthopedic procedures hinges on its cost-effectiveness, which requires more robust evidence [25, 26]. Until it can be definitively shown that robotic technology offers superior clinical outcomes and is financially viable, the integration of robotics into standard practice will face significant resistance. Furthermore, to substantiate claims of effectiveness, these benefits must be demonstrated through high-quality evidence, including data from multiple peer-reviewed publications.

A paramount obstacle is the substantial economic burden associated with these systems. This includes high capital acquisition costs, significant per-case consumable expenditures, and, where applicable, the additional costs of preoperative imaging (e.g., CT scans). These financial implications extend beyond individual institutions, impacting third-party payers and public health budgets.

Furthermore, the clinical utility of robotic technology—specifically regarding implant survivorship and functional outcomes—must be clearly established to justify the expense. However, platform heterogeneity regarding performance, accuracy, and workflows poses a challenge to the generalizability of findings and the standardization of surgical techniques.

Regional disparities further complicate adoption. A national geospatial study reported that only 2,571 of 34,216 orthopaedic surgeons in the United States had access to robotic knee arthroplasty systems, highlighting substantial geographic disparities in the availability of robotic technology. This inequity is compounded by differences in regulatory approval timelines and legal frameworks, which can impede the implementation of new programs and software updates [27].

Finally, the learning curve and training infrastructure remain critical barriers [28]. Surgeons require access to comprehensive training facilities, including cadaveric laboratories and mentorship programs. Proficiency maintenance necessitates continuous medical education. Without a robust infrastructure for skills acquisition and verification, the safe and effective integration of robotics into routine practice will be compromised.

In summary, the interplay of high economic costs, equivocal evidence on cost-utility, and disparities in training and access contributes to the uneven adoption of robotic technology. Addressing these multifaceted challenges requires rigorous evidence generation and global stakeholder collaboration.

#### Does robotic-assisted arthroplasty result in more precise implant positioning?

##### Recommendation

Robotic-assisted arthroplasty significantly improves the accuracy of implant positioning and alignment, demonstrating a marked reduction in radiographic outliers compared to conventional manual techniques.

##### Delegate Vote

Delegate vote: Agree 98%, Disagree 0%, Abstain 2%.

##### Justification

Several studies have demonstrated that robotic-assisted total knee arthroplasty (TKA) results in fewer outliers compared to conventional TKA, specifically in hip-knee-ankle (HKA) axis and implant positioning [29–41]. While the enhanced accuracy of robotic systems is well-documented, the translation of this radiographic precision into superior patient-reported outcome measures (PROMs) remains inconsistent [42–48]. Current systematic reviews demonstrate mixed results, indicating that radiologically superior alignment does not invariably equate to improved clinical function or pain scores in the short to medium term. A recent systematic review of randomized controlled trials encompassing 364 hips demonstrated no statistically significant difference in revision rates between robotic-assisted and conventional THA (RR 1.33, 95% CI 0.08–22.74) [49].

Furthermore, the utility of robotic precision is currently being re-evaluated in the context of emerging individualized alignment philosophies, which challenge traditional mechanical neutral targets. Consequently, high-quality longitudinal studies are imperative to definitively establish the correlation between robotic-mediated accuracy and long-term endpoints, including functional survivorship and patient satisfaction.

#### What are the recommended training prerequisites for surgeons prior to adopting robotic systems? Is proficiency in traditional manual surgery mandatory?

##### Recommendation

Prior to utilizing robotic-assisted surgery systems, surgeons should complete a structured training curriculum that encompasses theoretical education, computer simulation, and hands-on cadaveric workshops. Furthermore, proficiency in conventional manual arthroplasty is recommended as a prerequisite.

##### Delegate Vote

Delegate vote: Agree 91%, Disagree 7%, Abstain 2%.

##### Justification

Surgeons who are new to robotic systems must have sufficient experience with traditional manual surgery to effectively handle complications that may arise during robotic-assisted procedures. Studies have shown that robotic surgery, while offering high precision and potential for improved outcomes, cannot replace the core surgical skills required to manage unexpected challenges [50]. Surgeons without adequate traditional surgery experience may be less prepared for these situations, which could result in patient safety concerns.

The learning curve for robotic-assisted surgery is well-documented and varies depending on the complexity of the robotic system and the surgeon’s prior experience. It has been observed that surgeons who start with a robust foundation in manual surgical techniques tend to have faster and more successful transitions to robotic systems [51]. The initial use of robotic systems often increases operative time, and proficiency is typically achieved after 15–30 cases, depending on the surgical procedure and the surgeon’s background.

The importance of simulation and cadaver labs in the training process cannot be overstated [52]. Surgeons who undergo these hands-on training methods are more likely to acquire the dexterity and spatial understanding required to effectively use robotic systems. Simulation allows surgeons to practice techniques without the pressure of performing live surgeries, thus enhancing their readiness for actual clinical cases.

The field of robotic surgery is evolving rapidly, and surgeons need to stay current with advancements in technology, robotic systems, and new surgical techniques. Continuous education through workshops, conferences, and case reviews is critical to maintaining high standards of practice. This allows surgeons to refine their skills, address new challenges, and ensure that their knowledge of robotic surgery remains up to date [53].

Not all robotic systems are the same. Some platforms are more user-friendly, while others may have a steeper learning curve [54]. Surgeons need to be trained specifically for the robotic systems used in their practice, as compatibility with the system will affect their proficiency. This reinforces the need for training programs that are tailored to specific robotic platforms and surgical procedures.

#### Should robotic-assisted surgery be distinctly identified within joint arthroplasty registries?

##### Recommendation

We recommend that national joint arthroplasty registries mandate the recording of robotic assistance, ideally stratified by specific platform type. This distinction is critical to enable granular post-market surveillance and comparative effectiveness research.

##### Delegate Vote

Delegate vote: Agree 86%, Disagree 7%, Abstain 7%.

##### Justification

The distinct identification of robotic-assisted procedures within national registries is paramount for the rigorous evaluation of this evolving technology.

First, given the significant heterogeneity among robotic platforms (e.g., image-based vs. imageless; active vs. semi-active systems), generic coding (simply “robotic” vs. “conventional”) is insufficient. Stratified data collection enables the identification of system-specific failure modes or learning curve-associated complications unique to individual systems [6, 54, 55]. The urgency of this recommendation is underscored by recent findings from the AJRR, where the absence of robotic usage data in ~ 60% of TKAs led to significant data attrition and potential selection bias [56].

Second, separate stratification is essential for generating Real-World Evidence (RWE) to support both post-market surveillance and health economic analyses. Unlike controlled clinical trials, registries provide the scale needed to detect outlier performance and evaluate long-term survivorship. Furthermore, granular data enables healthcare systems to determine whether the substantial capital expenditure of specific robotic platforms translates into tangible improvements in revision rates or patient-reported outcomes (PROMs), thereby defining their true cost-effectiveness [57, 58].

### Statements of Group 2: Robotic-assisted total hip arthroplasty

#### Does robotic-assisted THA improve short-term outcomes?

##### Recommendation

Current evidence does not demonstrate a significant improvement in short-term outcomes for robotic-assisted THA compared to conventional techniques.

##### Delegate Vote

Delegate vote: Agree 77%, Disagree 21%, Abstain 2%.

##### Justification

It was found that robotic-assisted THA (rTHA) generally results in a shorter average length of hospital stay compared to conventional manual THA [59–62]. However, interpreting these results requires caution. A significant confounding factor is the variability in discharge criteria across different regions and institutions. While two small-scale studies showed conflicting results, a larger-scale study demonstrated a significantly shorter LOS in robotic-assisted groups compared to traditional manual surgery [61]. Another study indicated that conventional THA was an independent risk factor for hospital stays exceeding two days [62]. Across multiple studies, robotic THA was associated with a reduction of approximately 1.2 to 3 days in average LOS. However, the clinical significance of this finding may vary across countries due to socioeconomic differences. Interpretation of LOS differences is confounded by substantial regional and institutional variations in discharge criteria, reimbursement models, and cultural expectations regarding postoperative recovery.

Regarding functional outcomes, the literature presents mixed results. The majority of high-level evidence currently suggests comparable clinical outcomes between the two techniques. A systematic review including seven articles and 658 patients indicated that most studies found no significant difference in Patient-Reported Outcome Measures (PROMs) between robotic-assisted and conventional THA at a minimum of two years follow-up[63]. This finding is supported by a prospective cohort study, which reported no statistically significant differences in Oxford Hip Scores or UCLA activity scores at three years, despite the robotic group achieving better radiographic accuracy [64]. Similarly, other studies have supported these findings, showing no significant advantage in general patient-reported outcomes with robotic assistance [49, 65].

However, some evidence indicates potential benefits. A recent systematic review identified a trend toward higher postoperative scores in robotic cohorts across five studies, although this did not consistently reach statistical significance [66]. In contrast to the general trend, a propensity score-matched study by Clement et al. (*n* = 120) found that at one-year follow-up, the Forgotten Joint Score (FJS) was significantly higher in the robotic-assisted group compared to the conventional group, representing a clinically meaningful difference [67]. While satisfaction rates were also higher in the robotic-assisted group, this specific difference was not statistically significant.

In summary, while robotic assistance consistently improves component positioning, current evidence does not consistently show that this accuracy leads to superior short-term clinical outcomes.

#### Is the infection rate increased in robotic-assisted THA in comparison with manual surgeries?

##### Recommendation

Current evidence does not support the hypothesis that robotic-assisted THA is associated with an increased rate of infection compared to conventional manual techniques.

##### Delegate Vote

Delegate vote: Agree 79%, Disagree 14%, Abstain 7%.

##### Justification

The incidence of infection following THA is a critical factor that must be considered when adopting any new surgical technology, especially considering the advancements in robotic technology and its growing use in orthopedic surgeries. However, the current body of research presents limited findings, leading to uncertainty regarding whether robotic-assisted THA increases the infection rate compared to manual surgeries.

Most studies comparing conventional and robotic-assisted THA have primarily focused on component positioning, offset, leg length discrepancy, and functional outcomes [68–71]. However, few studies have reported differences in infection rates between these two groups.

One of the currently considered potential reasons for the increased incidence of periprosthetic joint infection (PJI) in robotic-assisted THA compared to conventional THA is the longer surgical time. The most recent systematic review of this issue showed that there is a definite association between longer operative time and infection rate [72], but that review included many underpowered studies with variable cutoff times for what was considered “long”. Additionally, it is important to recognize that most studies included in this article reflect a correlation between the two, but do not establish a causal relationship. Moreover, Robotic-assisted THA typically requires more surgical time than traditional THA during the initial stages of the learning curve. However, after surpassing the learning curve, the surgical times appear to be similar to those of conventional THA [73]. Therefore, the longer surgical time may not be attributable to the use of robotic-assisted technology itself, but rather to the surgeon’s familiarity with the technique.

Another potential reason widely considered is the additional equipment required in robotic-assisted THA, such as checkpoint pins, reference arrays, and imaging modalities/cameras, which are not needed in conventional THA. Moreover, the increased number of staff in the operating room to manage the robotic assistance may also contribute to the infection risk. However, no studies have conclusively identified these factors as definitive contributors to increased infection rates. Moreover, these issues can be significantly mitigated through the optimization of surgical team coordination and operating room sterilization and supply management processes.

LaValva SM et al. [74] performed propensity matching from a pool of 12,726 patients who had undergone THA. 8,022 who had undergone conventional THA. 2,678 (21%) had undergone computer navigation THA(CN-THA) and 2,026 had undergone robotic-assisted THA (RA-THA). They found that CN-THA and RA-THA were associated with longer operative times compared with conventional THA by 3 and 11 min, respectively. The rates of PJI after conventional THA (0.2% to 0.4%) were similar to those after CN-THA (0.4%) and RA-THA (0.4%). On the basis of logistic regression, the development of PJI was not associated with the use of computer or robotic assistance in THA.

Therefore, taking everything into account, we cannot conclude that the use of Robotic-assisted THA increases the incidence of PJI.

#### Can robotic-assisted hip replacement surgery reduce the incidence of postoperative joint dislocation?

##### Recommendation

Robotic-assisted total hip replacement (rTHA) surgery does not significantly reduce postoperative joint dislocation rates when compared to conventional manual approaches. Whilst there is clear evidence that rTHA may improve the surgical precision of component placement, the safe zone target in each patient remains undefined, and postoperative joint dislocation is multifactorial.

##### Delegate Vote

Delegate vote: Agree 93%, Disagree 7%, Abstain 0%.

##### Justification

Eleven articles are included in the generation of this review. The articles comprise 7 systematic reviews and meta-analyses, and 4 cohort studies with the levels of evidence between II and III.

Best available current evidence remains mixed and contentious as to whether robotic-assisted total hip arthroplasty (rTHA) reduces the incidence of postoperative joint dislocation compared to manual instrumentation total hip arthroplasty (mTHA). Various systematic reviews have assessed the impact of rTHA on different outcomes compared to results achieved by mTHA.

Han et al. (2019), in a meta-analysis of comparative studies involving 14 trials, showed an increased number of postoperative complications, including dislocations, in the robotic group compared to the manual group [75]. They questioned the rationale of robotic assistance in improving stability following total hip arthroplasty (THA).

In another comprehensive meta-analysis by Ng et al. (2021), 17 studies encompassing over 1000 patients were analyzed, comparing outcomes of rTHA with mTHA [76]. An increase in precision of acetabular component positioning was confirmed statistically (*p* < 0.001), but the dislocation rate did not differ significantly between the two groups. Likewise, Sweet et al. (2021) identified seven studies with 658 patients and demonstrated that the postoperative dislocation rate was not significantly different between the two techniques [77]. This lack of difference is notable since it implies that, despite benefiting from robotic assistance in the precision of the prosthesis component placement, there is no decrease in dislocation risk. The likely reason for this includes the multifactorial causation of THA instability, which goes beyond component alignment alone. Additionally, currently, there remains an inability to define the ideal component alignment target or safe zone in each patient. Therefore, whilst robotics adds precision, they do not necessarily increase accuracy.

Rowan et al.’s (2018) systematic review highlighted the multiple factors that may affect the post-THA dislocation rate [78]. They described different general and individual patient risk factors such as obesity and age, and whilst robotic-assistance and surgical approach might be beneficial, it was not sufficient to reduce the risk significantly. Similarly, Wright-Chisem et al. (2022) highlighted the progressive development of THA instability prevention strategies but noted that the use of safe zones for the acetabular component has failed to adequately mitigate instability [79].

Several papers have established some benefits of rTHA. Ogilvie et al. (2022) found that individual component positioning with the help of robotic systems could potentially decrease the level of dislocation in spinopelvic imbalance patients; however, there is no strong data to substantiate the advantage [80]. Although robotic assistance can enhance the precision of component positioning in THA, accurate planning and adaptation to variations in individual patient anatomy are relevant and remain to be defined. Di et al. (2024) analysed dislocation rates following THA using several variables, including surgical approach, robotic assistance, and computer-guided navigation, fluoroscopic guidance, and dual mobility bearings. They concluded that robotic assistance and fluoroscopic guidance may lower dislocation rates compared to conventional methods, although dislocation rates are not specified in the study [81]. In a review and meta-analysis by Kumar V et al. (2023), the effectiveness of rTHA was evaluated against manual techniques [82]. Analysing 17 studies encompassing 3,600 cases revealed that while robotic assistance improves component positioning precision, it does not significantly improve complication rates or functional outcomes. The authors concluded that though rTHA can reduce limb length differences and enhance precision, it does not notably impact dislocation rates in comparison to manual techniques.

Marcovigi A et al. (2022) assessed the risk of dislocation following rTHA across the three different surgical approaches: anterior, lateral, and posterior in 1,059 patients [83]. They found a low dislocation rate of 0.28% at 6-month follow-up, irrespective of surgical approach. Notably, the anterior approach demonstrated improved precision in cup positioning, thereby potentially lowering the risk of dislocation. However, no comparator group using a conventional technique was included in the study.

In their 2022 review, Chen ZP and others examined the effectiveness of CT scan-guided robot-arm THA in 24 studies, showing that rTHA delivers superior component placement, fewer errors with leg-length, and fewer cases of dislocation [84]. There was a trend toward a lower incidence of dislocation in the rTHA as compared to manual methods, but this was not statistically significant. Coulomb et al., in a matched-pairs analysis of 98 subjects, found an improvement in functional scores and reduced revision rate with rTHA [85]. Although the study did not explicitly state dislocation rates, the overall positive outcomes of the study for rTHA suggest a potential reduction in dislocation risk.

Robotic-assisted total hip replacement has not been proven scientifically to lead to a decrease in post-operative joint dislocations as compared to traditional methods. This is likely to be due to several confounding factors. These include the multiple factors that contribute and combine to increase dislocation risk. Additionally, the safe zone for an individual patient is probably quite specific and may be forgiving in some patients and narrow in others, depending on several interacting elements. The present literature is unanimous in recommending more high-quality research to address subtle issues affecting dislocation rates in THA and the true benefit of robotics in reducing the incidence of postoperative joint dislocation.

#### Is there any difference in the outcomes of THA with image-based and imageless robotic or navigation systems?

##### Recommendation

There is currently no high-level evidence suggesting a difference in short-term functional outcomes or revision rates between the two modalities. Image-based systems incur higher radiation exposure and procedural costs, whereas imageless systems offer a streamlined workflow but may exhibit slightly higher variability in outlier rates.

##### Delegate Vote

Delegate vote: Agree 93%, Disagree 7%, Abstain 0%.

##### Justification

Robotic systems for total hip arthroplasty (THA) can be categorized as image-based, intraoperative fluoroscopy-based, or imageless. Comparative studies and meta-analyses indicate that CT-based systems generally achieve mean errors of 2°–3° for cup abduction and anteversion, whereas imageless navigation systems typically have errors of 3°–4°, with anteversion discrepancies being more pronounced [86–88]. Image-based platforms require preoperative or intraoperative imaging, which increases radiation exposure and procedural cost, while imageless systems avoid radiation and reduce preoperative preparation time but may prolong intraoperative registration [89, 90]. In terms of clinical outcomes, Australian registry data showed no statistically significant difference in all-cause revision rates between Intellijoint navigation (1.8%), other navigation (2.2%), and conventional THA (2.2%) [91]. Japanese and Australian studies have reported that NaviSwiss can achieve less than 2° of angular error and less than 2 mm of leg length discrepancy in straightforward cases, although these series excluded severe deformities and lacked long-term follow-up [87, 92]. Overall, most available studies are single-center, small-sample, and retrospective, with some supported by system developers, raising potential conflicts of interest [86, 89]. Thus, while image-based systems appear more accurate, imageless systems provide advantages in cost, safety, and workflow efficiency, and there is no high-level evidence to support a difference in functional outcomes or revision risk; system choice should therefore be individualized according to surgeon experience, case complexity, and available resources.

#### Which patient populations are indicated for robotic-assisted primary THA?

##### Recommendation

Current evidence suggests that robotic-assisted THA is indicated for the same broad patient population as conventional THA. While no specific contraindications exist based on patient demographics, robotic assistance may offer distinct advantages in complex cases. Ultimately, the decision is driven by resource availability and institutional health economics rather than clinical exclusivity.

##### Delegate Vote

Delegate vote: Agree 84%, Disagree 14%, Abstain 2%.

##### Justification

A growing body of evidence suggests that robotic-arm-assisted total hip arthroplasty (rTHA) provides significant advantages in achieving precise implant positioning, particularly in challenging patient cohorts. Numerous studies have demonstrated that for patients with high Body Mass Index (BMI) or complex anatomical variations, robotic assistance overcomes the limitations of manual techniques by providing superior biomechanical accuracy and highly reproducible cup orientation [93, 94].

Crucially, current literature has not identified any specific patient populations for whom robotic-assisted surgery would be contraindicated or clinically inferior to conventional methods. On the contrary, data suggest that the majority of candidates suitable for primary THA can benefit from the enhanced intraoperative precision, better limb alignment restoration, and improved native biomechanics offered by these systems [77, 89, 95].

While rTHA has shown clear superiority in radiographic outcomes and some early reports indicate improved short-term functional recovery and gait kinetics, long-term clinical endpoints—such as rates of dislocation, infection, or revision—remain comparable to manual THA [96, 97]. Therefore, the primary barrier to the widespread adoption of robotic technology is not a lack of clinical applicability, but rather the significant economic burden. Institutional funding, high capital costs, and resource availability remain the decisive factors in the implementation of rTHA in clinical practice [67].

### Statements of Group 3: Robotic-assisted knee arthroplasty

#### What is the target difference between medial and lateral gaps during the soft-tissue balancing phase of robotic-assisted TKA?

##### Recommendation

In robotic-assisted total knee arthroplasty (RATKA), the target planned medial–lateral gap discrepancy after soft tissue balancing is suggested to be as follows: in extension, the lateral compartment gap should be no more than 1 mm greater than the medial compartment gap; in flexion (90°), the lateral compartment gap should be less than 3 mm greater than the medial compartment gap. This reflects the native knee’s ligament laxity pattern, where the medial side is tighter in extension, and the lateral side is relatively laxer in flexion

##### Delegate Vote

Delegate vote: Agree 79%, Disagree 16%, Abstain 5%.

##### Justification

In robotic-assisted total knee arthroplasty (RATKA), planned medial–lateral gap discrepancies are established by the robotic system based on preoperative planning and intraoperative assessments to replicate the native knee’s soft tissue balance. Typically, the planned target aims for approximately 1 mm greater lateral laxity than medial in extension, and less than 3 mm greater lateral laxity than medial in flexion (90°), reflecting the natural biomechanics where the medial side is tighter in extension and the lateral side relatively looser in flexion.

While these planned targets guide bone resections and soft tissue releases, it is important to recognize that actual gap measurements can change during surgery due to factors such as osteophyte removal, ligament releases, and trial component placement. Multiple studies, including recent work by Korean groups, demonstrate that medial and lateral gaps measured after bone resections may differ from the initially planned values, particularly affecting medial compartment gaps [98, 99]. Furthermore, gap balancing at mid-flexion remains challenging and is less frequently assessed intraoperatively [100].

There is no universal consensus on rigid numeric thresholds for final gap discrepancies or on which intraoperative time point should define the “acceptable” balance. The robotic system’s planned gap targets serve as a dynamic guide that must be interpreted in the context of evolving intraoperative findings and patient-specific factors such as deformity severity, joint stiffness, and soft tissue quality.

Current measurement tools, including robotic platforms, provide valuable quantitative targets but cannot fully capture soft tissue tension or dynamic ligament behavior, so clinical judgment remains essential [101, 102].

Thus, while the planned medial–lateral gap discrepancies outlined provide a practical framework for achieving balanced ligament tension during RATKA, flexibility in interpretation and adaptation is required during the surgical workflow.

#### Is the philosophy of personalized alignment technically achievable in robotic-assisted total knee arthroplasty?

##### Recommendation

Robotic-assisted systems serve as a definitive enabling technology that makes personalized alignment strategies technically achievable and reproducible.

##### Delegate Vote

Delegate vote: Agree 100%, Disagree 0%, Abstain 0%.

##### Justification

Robotic platforms have fundamentally altered the feasibility of personalized alignment by offering sub-millimeter control over osteotomy planes. By integrating preoperative CT or intraoperative morphometric data, robotics allows surgeons to precisely replicate the patient’s native constitutional alignment and joint line obliquity—a task often difficult to reproduce consistently with manual instrumentation [103, 104].

While the execution of personalized plans is now robustly supported by robotic systems [105, 106], the definition of optimal targets remains an evolving science. Challenges persist in establishing standardized boundaries for safe personalization and validating patient-centered outcomes. Furthermore, the integration of postoperative functional feedback into the planning loop is still in its infancy.

While real-time, dynamic, tension-based personalized alignment has not yet been fully realized, current robotic technologies have successfully bridged the gap between theoretical philosophy and clinical reality, rendering personalized alignment a practical standard of care for adopters [107].

#### In cases of conflict between achieving a specific target alignment and maintaining soft tissue balance, which should be prioritized in robotic-assisted TKA?

##### Recommendation

In robotic-assisted TKA, soft tissue balance should take precedence over rigid adherence to a specific alignment target. When conflict arises, it is recommended to adjust the implant alignment (within safe boundaries) to achieve optimal soft tissue tension, rather than performing extensive releases to force a predetermined alignment.

##### Delegate Vote

Delegate vote: Agree 91%, Disagree 9%, Abstain 0%.

##### Justification

Robotic-assisted total knee arthroplasty (RATKA) allows precise quantification of soft tissue balance before bone cuts through intraoperative planning. This enables surgeons to adjust osteotomy and implant positioning flexibly rather than strictly adhering to mechanical alignment (MA), thereby optimizing gap balance and minimizing the need for extensive soft tissue releases. Masilamani et al. [108] demonstrated in a bilateral robotic-assisted TKA study comparing MA and functional alignment (FA) that dynamic balancing via implant position modification effectively achieved well-balanced knees, particularly in the FA group.

Consistent with these findings, multiple studies [109–111] report that FA significantly reduces postoperative soft tissue imbalance compared to MA, which correlates with decreased soft tissue releases and improved early recovery. Furthermore, a series of investigations over the last five years indicates no significant differences in early pain relief, functional outcomes, or implant survivorship when additional soft tissue releases are avoided [112, 113]. These data collectively support prioritizing soft tissue balance over strict alignment restoration in RATKA, especially when these goals conflict.

Therefore, we recommend that during robotic-assisted TKA, soft tissue balancing should take precedence, and alignment restoration can be moderately compromised during planning and osteotomy to achieve optimal functional balance.

#### Does robotic assistance simplify the achievement of appropriate soft tissue balance in TKA?

##### Recommendation

Robotic assistance significantly simplifies the process of soft tissue balancing by enabling “pre-resection balancing.” This capability allows surgeons to virtually model component position and predict gap tension prior to any osteotomy, thereby minimizing the need for complex soft tissue releases.

##### Delegate Vote

Delegate vote: Agree 95%, Disagree 5%, Abstain 0%.

##### Justification

Robotic-assisted total knee arthroplasty (RA-TKA) offers a unique capability for pre-resection soft tissue assessment, allowing surgeons to virtually simulate bone cuts and component placement before any bone is actually cut. This facilitates refinement of surgical plans to better achieve targeted alignment and soft tissue balance.

Studies have demonstrated that robotic systems provide reliable and reproducible quantitative measurements of soft tissue laxity intraoperatively, with accuracy comparable to or better than manual methods. Clinical evidence shows that RA-TKA significantly reduces the need for additional soft tissue releases, indicating more precise and consistent soft tissue balancing [114–116].

Therefore, when combined with appropriate quantitative tools, robotic assistance enables surgeons to achieve appropriate soft tissue balance more easily and reliably than conventional techniques, contributing to improved surgical outcomes in knee arthroplasty.

#### What is the optimal strategy for pin placement in robotic-assisted TKA?

##### Recommendation

In robotic-assisted total knee arthroplasty (rTKA), both intra-incisional pins and extra-incisional pins generally yield comparable clinical outcomes in most patients. Nevertheless, particular caution must be exercised in high-risk populations to minimize the occurrence of associated complications.

##### Delegate Vote

Delegate vote: Agree 86%, Disagree 7%, Abstain 7%.

##### Justification

Tracker pin-site complications are a unique but infrequent sequela of robotic TKA, with a reported incidence of approximately 1.4% [117]. These include superficial infection, pin-tract fracture, and neurovascular injury.

While debate exists regarding intra- vs. extra-incisional placement, current evidence suggests comparable outcomes [118]. The critical factor is anatomic safety. For the femur, pins should be placed well proximal to the trochlea to avoid stress risers in the anterior cortex, which is a high-risk zone for fracture. For the tibia, medialized placement is advocated to safeguard the neurovascular bundle [119].

Bicortical fixation is generally recommended to maximize stability and prevent array loosening, particularly in patients with osteopenia or osteoporosis. Unicortical pins, while reducing drill depth, carry a higher risk of toggle and subsequent loosening in poor bone quality.

To minimize stress risers, multiple passes through the same cortex should be avoided. In high-risk populations (e.g., severe osteoporosis), careful consideration of pin diameter and spacing is essential.

#### Does rTKA reduce the rates of reoperation, revision compared to conventional manual TKA?

##### Recommendation

Limited evidence suggests that rTKA may reduce the rates of reoperation and revision compared to manual TKA (mTKA), and its advantages in these aspects may be more pronounced in complex TKA cases. Moreover, the long-term effects remain unclear.

##### Delegate Vote

Delegate vote: Agree 95%, Disagree 5%, Abstain 0%.

##### Justification

Recent studies have explored the clinical outcomes and complications of rTKA compared to mTKA, presenting a complex clinical landscape [120–123]. In a long-term retrospective study, there was no significant difference in readmission rates and overall adverse event incidence between rTKA and mTKA. However, patients in the rTKA group had shorter hospital stays and relatively lower rates of reoperation and Periprosthetic Joint Infection (PJI) [124]. Another study also showed that patients who underwent rTKA had significantly lower rates of deep vein thrombosis, myocardial infarction, and pulmonary embolism within 90 days postoperatively compared to those who underwent mTKA. However, there were no significant differences in revision rates and PJI rates between the two groups [125]. In another randomized controlled trial spanning over 10 years, researchers compared the clinical outcomes of rTKA with those of mTKA. The results showed that there were no significant statistical differences in key clinical indicators between the two surgical approaches, including functional outcome scores (*p* = 0.992), aseptic loosening, overall prosthetic survival rates (*p* = 0.972), and complication incidence rates (re-operation and infection rates) [126]. In summary, rTKA has shown positive clinical outcomes in terms of postoperative pain and hospital stay. However, its role in terms of reoperation rates, revision rates, and infection incidence remains unclear [127].

For complex TKA surgeries, rTKA may have an advantage in reducing re-operation rates, revision rates, and infection rates. A study conducted between 2017 and 2020 on 341 primary rTKAs divided patients into two groups: complex TKA (*n* = 218) and non-complex TKA (*n* = 123). The study found that robotic-assisted primary total knee arthroplasty significantly reduced the surgical time and hospital stay for complex TKA and controlled the 90-day complication and re-admission rates to the level of non-complex TKA [128]. These studies collectively suggest that while rTKA demonstrates potential advantages in certain short-term clinical metrics, its overall clinical value still requires further confirmation through large-scale, long-term prospective randomized controlled trials.

In summary, although rTKA is gaining clinical attention due to its technical advantages in precise bone preparation and accurate prosthesis positioning, the existing evidence has not yet conclusively demonstrated that it can significantly reduce the rates of reoperation, prosthetic revision, and surgical site infection.

#### Does robotic-assisted UKA result in improved alignment and reduced outliers compared to manual instrumentation, PSI, and computer navigation?

##### Recommendation

Robotic-assisted unicompartmental knee arthroplasty (UKA) demonstrates superior accuracy in component positioning and alignment compared to manual instrumentation and Patient-Specific Instrumentation (PSI). However, current evidence does not demonstrate a significant superiority of robotics over computer navigation systems in terms of alignment precision.

##### Delegate Vote

Delegate vote: Agree 84%, Disagree 12%, Abstain 4%.

##### Justification

Several systematic reviews have consistently demonstrated that robotic-assisted UKA, especially with systems such as Mako, improves the accuracy of implant positioning and limb alignment compared to manual techniques [129–132]. Although some studies have reported that PSI-UKA achieves accuracy comparable to that of robotic-assisted UKA (R-UKA) [133], a Bayesian analysis including 12 studies and 871 knees demonstrated that in the sagittal plane, R-UKA exhibited a significantly reduced deviation angle of the femoral component compared to PSI-UKA (MD: 4.16; 95% CI 0.21–8.07) [129]. Another meta-analysis based on 18 RCTs involving 1,564 knees finds that PSI is associated with a significantly higher incidence of femoral component malposition outliers when compared to both robotic-assisted and navigated techniques [134].

Currently, high-quality randomized controlled trials directly comparing robotic-assisted UKA with navigation-assisted UKA are extremely limited. Therefore, while rUKA offers measurable technical advantages over manual and PSI methods, dedicated high-quality comparative studies specifically in UKA are lacking, and its superiority over computer navigation systems remains unproven, warranting further investigation.

#### Does robotic-assisted UKA improve clinical outcomes compared to conventional techniques?

##### Recommendation

Robotic-assisted UKA improves surgical precision and reproducibility and is associated with lower revision rates and modest improvements in selected functional outcomes compared with conventional UKA. However, evidence remains insufficient to establish consistent superiority across all clinical outcome measures or patient-reported outcome domains.

##### Delegate Vote

Delegate vote: Agree 86%, Disagree 12%, Abstain 2%.

##### Justification

Regarding clinical outcomes, early randomized controlled trials with mid-term follow-up reported no significant differences in several commonly used functional scores between robotic-assisted and conventional UKA [135, 136]. However, more recent systematic reviews and meta-analyses incorporating larger patient populations have demonstrated modest but significant improvements in selected patient-reported outcome measures, including Knee Society Score (KSS) and Forgotten Joint Score (FJS) [137–139], together with lower complication and revision rates following robotic-assisted UKA [140, 141].

Evidence from national joint registries further supports a potential survivorship benefit [140, 141]. Data from the Australian Orthopaedic Association National Joint Replacement Registry (AOANJRR) demonstrated a significantly lower risk of revision for robotic-assisted UKA compared with conventional UKA in real-world practice [140]. Nevertheless, whether robotic assistance confers clinically meaningful superiority across all functional domains and patient populations remains uncertain, as high-quality long-term comparative studies remain limited.

Length of hospital stay (LOS) has shown inconsistent findings across studies and appears to be influenced more strongly by institutional discharge protocols, perioperative care pathways, and healthcare systems than by the surgical technique itself[142–145]. Therefore, current evidence does not support a consistent reduction in LOS attributable to robotic assistance alone.

## Discussion

In this expert consensus, we established 18 statements covering the general principles, total hip arthroplasty (THA), and total/unicompartmental knee arthroplasties (TKA/UKA) utilizing robotic assistance. Driven by rapid technological advancements, an increasing number of orthopedic surgeons are becoming highly reliant on robotic platforms in their routine practice. Experientially, many surgeons subjectively perceive that robotic assistance yields superior clinical outcomes—particularly noting heightened intraoperative confidence, enhanced execution of complex soft-tissue balancing, and simplified management of severe deformities. However, a critical gap exists between this growing clinical enthusiasm and current Evidence-Based Medicine (EBM). While robotic systems definitively eliminate radiographic outliers and deliver millimeter-level precision, objective, high-quality, long-term data demonstrating consistent superiority over conventional manual techniques—specifically regarding infection rates, dislocation, and long-term functional survivorship—remain insufficient and often conflicting. The current paradox is that technological capability has outpaced the generation of long-term longitudinal data.

## Conclusion

Robotic-assisted arthroplasty is a powerful enabling technology, yet it remains constrained by high costs, a distinct learning curve, and the indispensable prerequisite of manual surgical proficiency. Clinically, its greatest value lies in managing complex reconstructions and executing advanced strategies, such as personalized alignment and dynamic soft-tissue balancing. To reconcile high subjective surgeon satisfaction with the current lack of definitive long-term evidence, national registries should mandate platform-specific tracking. Future research should move beyond verifying radiographic precision to identifying specific patient phenotypes that derive tangible, long-term clinical and economic benefits.

## Data Availability

No datasets were generated or analysed during the current study.
